# Impact of a large-scale event on SARS-CoV-2 cases and hospitalizations in the Netherlands, carnival seasons 2022 and 2023

**DOI:** 10.1016/j.puhip.2024.100523

**Published:** 2024-06-27

**Authors:** Koen M.F. Gorgels, Nicole H.T.M. Dukers-Muijrers, Ymke J. Evers, Volker H. Hackert, Paul H.M. Savelkoul, Christian J.P.A. Hoebe

**Affiliations:** a Department of Sexual Health, Infectious Diseases and Environmental Health, Living Lab Public Health, South Limburg Public Health Service, PO Box 33, 6400 AA Heerlen, the Netherlands; b Department of Social Medicine, Care and Public Health Research Institute (CAPHRI), Maastricht University, PO Box 616, 6200 MD Maastricht, the Netherlands; c Department of Health Promotion, Care and Public Health Research Institute (CAPHRI), Maastricht University, PO Box 616, 6200 MD Maastricht, the Netherlands; d Department of Medical Microbiology, Infectious Diseases and Infection Prevention, Care and Public Health Research Institute (CAPHRI), Maastricht University Medical Centre (MUMC+), PO Box 5800, 6202 AZ Maastricht, the Netherlands

**Keywords:** COVID-19, Carnival, Hospitalizations, Population immunity, Preventive measures, Vaccination

## Abstract

**Objectives:**

The COVID-19 pandemic highlights the importance of understanding facilitators for disease transmission. Events such as Carnival, characterized by large gatherings and extensive social interactions, have the potential to become ‘super spreading events' for respiratory infections. This paper aims to assess the impact of large gatherings on virus transmission, providing crucial insights for the development of effective public health strategies.

**Study design:**

An ecological study was performed.

**Methods:**

The age-standardized number of COVID-19 cases reported in 2022, stratified by age (under 60 and 60+ years) was compared countrywide for Dutch provinces where Carnival was celebrated versus those where it was not. Additionally, we compared standardized hospitalization rates in 2022 and 2023 for both areas.

**Results:**

Countrywide, 2,278,431 COVID-19 cases were reported between 06-02-2022 and 10-04-2022. Daily incidence increased after Carnival, peaking at 803 per 100,000 inhabitants for under 60s in carnival provinces and 368 in non-carnival provinces. For individuals 60+ daily incidence peaked at 396 in carnival provinces and 247 in non-carnival provinces. Over the 10 weeks following the start of Carnival, the carnival provinces demonstrated a 15 % (2022) 17 % (2023) higher hospitalization rate compared to non-carnival provinces.

**Conclusions:**

The peak in cases and hospitalizations in regions actively celebrating Carnival compared to the rest of the Netherlands qualifies Carnival as a ‘super-spreading’ event. Our findings underscore the elevated risk of respiratory infections associated with large gatherings, advocating guided policies, including transparent risk communication and healthcare preparedness.

**What this study adds**.

The COVID-19 pandemic has underscored the urgency of understanding transmission dynamics and effective disease control. Daily COVID-19 incidence across all age groups and hospitalizations due to COVID-19 increased in regions celebrating carnival when compared to regions that did not celebrate carnival. During the 10 weeks following the start of Carnival, the carnival provinces demonstrated a 15 % (2022) to 17 % (2023) higher hospitalization rate compared to non-carnival provinces. This study stands out for its combination of COVID-19 infection rates and hospitalization data, diverging from the typical focus solely on infection rates. Additionally, to our knowledge, it's the only research to assess COVID-19 infections against a significant backdrop of acquired immunity.

**Implications for Policy and Practice**.

Our findings imply that carnival and similar large scale events can be characterized as a super-spreading event, given its discernible impact on both COVID-19 transmission and hospitalizations. For upcoming pandemics, thoughtful consideration of supplementary measures, such as event cancellations, becomes crucial for effectively mitigating transmission risks, especially in the initial stages when immunity is low.

## 1 Introduction

The COVID-19 pandemic has highlighted the need to better understand what drives transmission and disease burden and what is effective in control of infectious diseases [1]. Large-scale events, characterized by mass gatherings and extensive social interactions, have gained attention as potential catalysts or ‘super spreading events’ for viral transmission and can contribute to variant predominance [[2], [3], [4], [5]]. Carnival, a vibrant and culturally significant Christian event celebrated in numerous countries, stands as a notable example of a mass gathering with numerous and close social interactions. However, research examining the impact of carnival on hospitalization rates remains limited. Most studies have focused primarily on incidence rates, with only one study on influenza identifying an increase in hospitalizations following this event [6].

Carnival festivities typically involve multi-day parades, indoor and outdoor parties, and communal gatherings that attract local residents, visiting friends and family, and tourists. These events create an environment conducive to close physical contact and potential transmission. In Germany, the first major outbreak of COVID-19 has been associated with Carnival, while in the Netherlands in 2020, regions where Carnival is traditionally celebrated experienced a higher number of COVID-19 cases compared to other areas [6,7]. Additionally, the first outbreak in a long-term care facility in the Netherlands was connected to activities related to German Carnival and a large serology study established a dose-response relationship between duration of celebrating carnival and SARS-CoV-2 seropositivity [8,9]. Understanding the relationship between these large-scale events and the transmission dynamics of the virus is crucial for developing effective public health strategies.

In the Netherlands, Carnival is mainly celebrated in the country's southern regions, due to a historical schism of Christianity in the 16th Century. Protestantism became the dominant religion north of the rivers Meuse and Rhine, the Catholic religion remained dominant in the southern parts of the country, an area congruent with today's provinces of Limburg and North Brabant. While Carnival celebrations take place on a limited scale in other parts of the country, massive Carnival festivities pervading public life for their duration remain predominant in the southern provinces of Limburg and North Brabant. This study aims to examine the impact of Carnival on COVID-19 transmission and hospitalization rates by analyzing publicly available data from 2022 to 2023. Specifically, our study focuses on comparing the primary Carnival regions of Limburg and North Brabant with the rest of the country. The goal is to assess transmission dynamics associated with this cultural event and its potential effects on the spread of COVID-19 to inform potential risk communication efforts and mitigating measures by public health authorities for Covid-19 but might also serve as a model for other respiratory viruses.

## 2 Methods

### 2.1 Setting

#### 2.1.1 Carnival study periods

Carnival is culturally based in Catholicism, a significant and dynamic celebration for local communities and prominently observed in the southern provinces of the Netherlands. This days-long celebration is characterized by parades of people dressed up in elaborate costumes, and massive energetic indoor and street parties. Traditionally aligned with the period preceding Lent, carnival signifies a time of festivity and mirth preceding the onset of the contemplative season. We studied two seasons of Carnival in 2022 and 2023. In 2022, the officially recognized carnival dates spanned from February 26 to March 1, while in 2023, the festivities occurred from February 18 to February 21. Additionally, in the weeks leading to carnival various pre-carnival activities occur.

#### 2.1.2 Differentiating carnival and non-carnival regions

We regrouped regions in the Netherlands as carnival provinces (Limburg and Brabant) and non-carnival provinces. Thereby, we discarded small municipalities within non-carnival provinces that celebrate carnival. Factors such as daily commuting, social interactions, and economic activities contribute to the spread of the virus beyond carnival events. By focusing on large provinces, we gain a comprehensive understanding of the impact of carnival on COVID-19 transmission. This approach allows for an assessment of the broader regional impact and avoids the potential confounding influence of transmission extending beyond specific carnival celebrations in small municipalities. Furthermore, from a practical standpoint, implementing potential mitigating measures is usually conducted at a provincial level therefore increasing the practical applicability of our results.

#### 2.1.3 Infection prevention measures during our study periods

The general advice during our study period in 2022 was to conduct a self-test when developing COVID like symptoms and have a positive test confirmed by RT-PCR. This recommendation was dropped on April 11, 2022, so no representative data on COVID-19 cases is available for 2023. On February 25, 2022, the day prior to the official commencement of carnival, several long-standing preventive measures were completely relaxed. These measures included the removal of the mandatory 1.5-m social distancing requirement, the discontinuation of the corona entry ticket requirement for the hospitality industry, and the reinstatement of normal opening hours. Medical mouth masks were only required in public transportation and for specific large inside events. This was not the case for carnival events and activities, leading to a kind of natural experiment as almost all mitigating measures were lifted just before the carnival season started. In 2023, there were no restrictions whatsoever during our study period. During our study period in both 2022 and 2023 subvariants of the omicron variant were dominant [10].

#### 2.1.4 Descriptive analyses

The baseline comparison between carnival and non-carnival regions encompassed several characteristics. These included demographic factors such as total population, population density, and age distribution. Notably, we expressed the latter as the percentage of individuals aged 60 years or older, a demographic that constitutes the predominant portion of hospital admissions [11]. Individuals had completed a primary vaccinations scheme if they had at least two rounds of vaccinations of Pfizer-BioNTech (Pfizer), Spikevax (Moderna) or Vaxzevria (AstraZeneca) or one Jcovden (Janssen) vaccination. Booster shots of Pfizer-BioNTech or Spikevax were introduced in November 2021, initially for individuals aged 60 and above and specific risk groups, later expanded to all adults above 18.

#### 2.1.5 Statistical analyses

Publicly available data from the RIVM were used in the analyses. Given the transmissibility of COVID-19, the current infection rates in each province are intricately tied to past rates. To ensure an equal starting point for observations, a *t*-test was conducted comparing the number of cases and hospitalizations between provinces celebrating Carnival and those that do not, specifically in the week preceding Carnival. Data were expressed as number of cases per 100,000 inhabitants or number of hospitalizations per 1,000,000 inhabitants. The 7-day rolling average of new cases per 100,000 inhabitants for individuals younger and older than 60 years old or hospitalizations were plotted with calendar time (weeks). For hospital admissions, we also calculated present a 95 % confidence interval under the assumption of a Poisson distribution. All statistical analyses were performed with R statistical Software (R Foundation for Statistical Computing, Vienna, Austria).

## 3 Results

In total, both provinces celebrating carnival comprise a population of 3,711,176, while the non-carnival provinces have a population of 13,879,496, with similar population densities (reference date 1-1-2022). The carnival provinces have a higher proportion of individuals aged 60 years and older (29.3 % versus 26.1 %) and also exhibit higher vaccination rates among individuals in this age group, both for the primary and the booster rounds (Table 1).

**Table 1:** Baseline characteristics of both Carnival and non-Carnival regions *Reference date 1-1-2022 **Reference date 21-2-2022.

	Carnival provinces	Non-carnival provinces
Population (n)*	3,711,176	13,879,496
Population/km^2*	523	520
Percentage population 60 years or older (60+) *	29.3 %	26.1 %
Percentage 60+ with complete primary vaccination **	92.9 %	90.4 %
Percentage 60+ with booster vaccination **	84.4 %	81.1 %

**Fig. 1:**
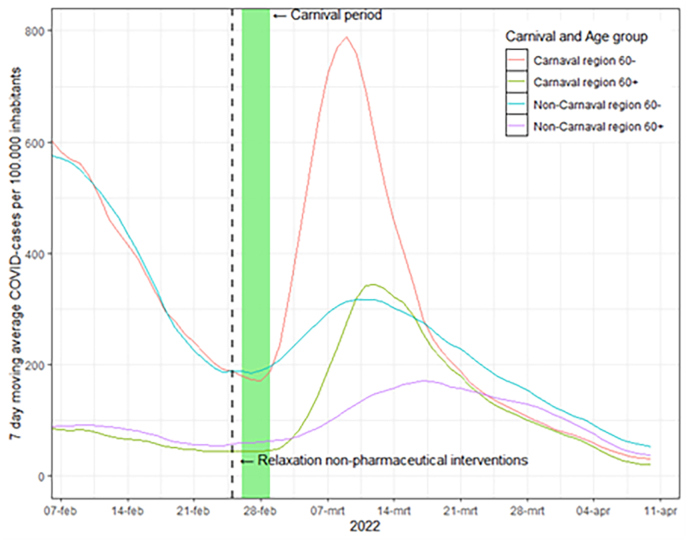
COVID-19 cases per age group.

Countrywide, a total of 2,278,431 COVID-19 cases were reported between 06-02-2022 and 10-04-2022. A *t*-test comparing the 7-day moving average pre-Carnival (on February 25, 2022) revealed no significant difference between the carnival provinces and non-carnival provinces (p-value = 0.26). The COVID-19 incidence per 100,000 inhabitants rapidly rose after the carnival event in both carnival and non-carnival areas in both age groups. The 7-day moving average of reported cases reached its peak for individuals younger than 60 in carnival provinces on March 9 (n = 803), for individuals younger than 60 in non-carnival provinces on March 10 (n = 368), for individuals aged 60 years or older in carnival provinces on March 12 (n = 396), and for individuals older than 60 in non-carnival provinces on March 17 (n = 247) (Fig. 1).

**Fig. 2:**
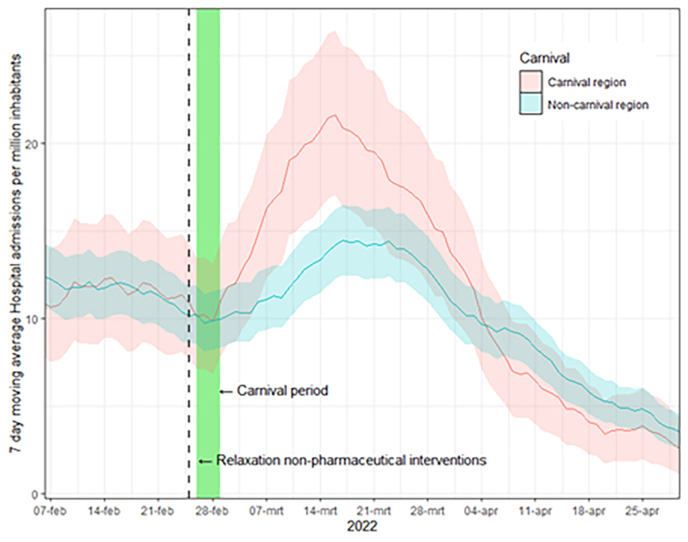
Hospital admissions following carnival 2022.

Between February 6 and April 30, 2022, there were a total of 16,373 reported hospitalizations. A *t*-test comparing the 7-day moving average of hospital admissions pre-carnival (on February 25, 2022) revealed no significant difference between carnival and non-carnival provinces (p-value = 0.62). After carnival hospital admissions rose both in carnival and in non-carnival provinces. The peak of hospital admissions occurred on March 16, with a value of 21.6 (per 1,000,000 inhabitants) in the carnival provinces and on March 17, with a value of 14.7 in the non-carnival provinces (Fig. 2). When examining the aggregated data on a weekly basis, an increase of 47–54 % in hospital admissions was observed 2–3 weeks after carnival (supplementary material). Notably, starting from week 14, the number of hospital admissions decreased in the carnival regions. Throughout the 10-week period following carnival, the carnival provinces experienced a 17 % higher rate of hospital admissions compared to the non-carnival provinces. When analyzing the cumulative hospital admissions, it was observed that carnival and non-carnival provinces reached an equal number of admissions in July (supplementary material). Afterwards, the number of hospital admissions rose faster in non-carnival provinces compared to carnival provinces.

**Fig. 3:**
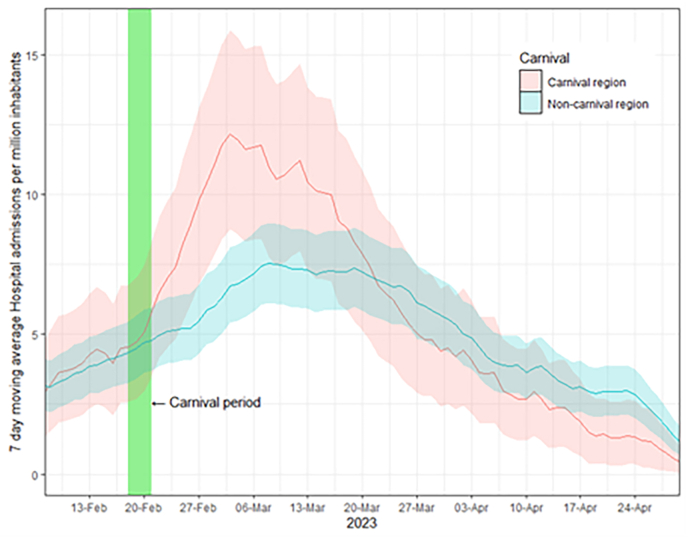
Hospital admissions following carnival 2023.

Between February 6 and April 30, 2023, there were a total of 7225 reported hospitalizations. Hospital admissions rose after carnival in both carnival and non-carnival provinces. The peak of hospital admissions occurred on March 3 (12.2), in the carnival regions and on March 8 (7.5), in the non-carnival regions (Fig. 3). When examining the aggregated data on a weekly basis, an increase of 52–71 % in hospital admissions was observed in the weeks after carnival (supplementary material). Notably, starting from week 12, the number of hospital admissions decreased in the carnival provinces. Overall, during the 10-week period, including the week of carnival, there was a 15 % higher number of hospital admissions in the carnival provinces compared to the non-carnival provinces. Beyond May, the cumulative hospital admissions for both types of provinces regions did not align (supplementary material).

## 4 Discussion

Our study reveals that areas in the Netherlands which celebrated Carnival in 2022 and 2023 experienced an increase surge in COVID-19 cases and hospitalizations that was higher than the increase observed in the rest of the country. This increase was followed by a decline about 4–5 weeks post-carnival. These results underline the heightened risk of respiratory infections linked with large gatherings and underscore the need for guided policies, including clear risk communication to potential attendees and increased hospital preparedness.

During the peak period, the incidence of COVID-19 cases in carnival regions was approximately 1.5–2 times higher compared to the rest of the Netherlands, which aligns with findings from a German study conducted in Cologne, a renowned carnival stronghold in Germany [12]. The confluence of heightened social interactions and recently relaxed preventive measures likely played a role in the increased transmission of COVID-19, as evidenced by previous research [13]. Furthermore, the occurrence of indoor events with inadequate ventilation likely exacerbated the situation, as multiple outbreaks in nightclubs have been documented [[14], [15], [16]]. Similarly, a previous Dutch study from 2020 reported a similar difference in incidence rates between carnival regions and non-carnival regions, although testing restrictions and possible differences in baseline conditions may have influenced these findings back in 2020 [6]. However, seroprevalence of antibodies was found to be higher among individuals who participated in carnival festivities, with the extent of seropositivity correlating with the duration of their engagement in indoor carnival activities [8]. Lastly, a study conducted in the Canary Islands demonstrated that the cancellation of carnival celebrations due to a sandstorm resulted in significantly fewer COVID-19 cases [17].

The overall rise in the number of COVID-19 cases across the entire Netherlands can be attributed, in part, to the lifting of infection prevention measures just before carnival. Across the entire Netherlands, physical distancing of 1.5 m was no longer mandated, the corona entry ticket requirement for the catering industry was discontinued, and normal opening hours were reinstated. The date of the peak of COVID-19 cases among individuals younger than 60 was similar across all regions, indicating increased overall transmission with an apparent further increase in carnival regions.

Compared to the rest of the country, we observed an earlier peak of COVID-19 cases among individuals aged 60 years and older. This suggests that older age groups participated in Carnival activities. Carnival celebrations often attract people of all ages, including older individuals who may have a long-standing tradition of participating in these cultural events. However, it is noteworthy that the peak among individuals aged 60 years and older was slightly delayed compared to the peak in younger individuals, suggesting some degree of secondary transmission outside of carnival festivities, likely influenced by factors such as intergenerational interactions and community spread. Our earlier paper on COVID-19 transmission following reopening of the hospitality sector after a prolonged lockdown also saw evidence for secondary transmission from younger individuals to the older population [18].

In 2022 and 2023, carnival regions exhibited a 15–17 % rise in hospital admissions compared to non-carnival regions in the 10-week period following the start of carnival. The difference between both regions on a weekly basis reached 54–71 % 1–2 weeks after carnival, highlighting the impact of carnival impact on healthcare resources. Notably, a rebound effect was observed in both years, with a decline in hospitalizations occurring approximately 4–5 weeks after carnival's conclusion. This trend aligns with findings from a Dutch influenza study, indicating post-peak declines in hospitalizations [6].

The observed ‘rebound effect’ can probably be attributed to a decrease in susceptible individuals within the population [19]. The initial surge in cases led to a higher proportion of people who had recovered from COVID-19 and developed immunity. As a result, the population of susceptible individuals diminished, leading to a decrease in both COVID-19 cases and hospitalizations. However, it is important to note that complete herd immunity was not achieved, and a year later, a new surge in cases was observed, which aligns with findings on waning immunity [20].

The lower number of cumulative hospital admissions in carnival regions from July 2022 onwards is unlikely to be solely due to the rebound effect of carnival events four months prior. Other factors, including increased vaccination rates, may have played a role in the relative decline of hospital admission in carnival regions. In contrast to 2022, no similar pattern was observed in 2023. Further research focusing on vaccination coverage and its relationship with hospitalization trends would provide valuable insights. However, this study's scope and objectives do not include such analysis.

After carnival in 2023, hospital admissions increased throughout the Netherlands, even in the absence of any relaxation of measures. While it is possible that spillover effects from carnival contributed to this rise, it is important to recognize that hospitalizations had already started to increase prior to carnival in both carnival and non-carnival regions. This suggests that other factors, such as the emergence of new circulating (sub)variants, may have also played a role. Furthermore, following the relaxation of measures, the Netherlands experienced multiple periods of increased rates of infections in all parts of the country [21]. Lastly, the number of hospitalizations in 2023 was lower than in 2022, likely due to increased immunity and higher vaccination rates.

Our findings have broader implications for the transmission dynamics of COVID, indicating that large-scale events such as carnival can contribute to increased transmission. This pattern has been observed in other events, even in the presence of restrictions, like the Olympic Games in Japan, spring break celebrations, and holiday gatherings around the 4th of July [[22], [23], [24], [25], [26], [27]]. This highlights the importance of comprehensive risk assessments and guided policies, including potential event cancellations during periods when healthcare facilities are nearing capacity. Additionally, healthcare systems must be prepared for potential surges in COVID-19 hospital admissions and the challenges of increased work absence due to illness or need to quarantine, emphasizing the need for proactive planning to ensure quality care delivery during large-scale events and future pandemics.

It is important to note that carnival in 2022 and 2023 took place against a backdrop of accumulated immunity resulting from prior infections and vaccinations. This highlights the interplay between population immunity and the impact of large-scale events. An illustrative example is seen in June 2021 when most restrictions in the Netherlands were briefly lifted, only to be reinstated two weeks later due to an alarming almost twenty-fold increase in incidence [18]. This emphasizes the potentially magnified effect of events like carnival in populations with limited prior exposure and immunity which can occur during early stages of future pandemics.

Our research also sheds light on the potential spread of other respiratory infections, such as influenza and pneumococcal disease, during carnival as an earlier modelling study calculated that mass gatherings prior to the endemic peak can increase the peak prevalence and overall attack rate [5]. One study even observed a surge in influenza hospitalizations post-carnival [6]. In light of these findings, we advocate for individuals, especially the elderly or those with weakened immune systems, to be provided with informed choices about attending carnival. Considering the cultural significance of carnival for local communities, strict measures should be considered only as a last resort.

This study's strength lies in its use of large datasets and the year-over-year comparison of incidence and hospitalization rates. However, our study is not without limitations. Although no large demographic differences were observed between carnival and non-carnival regions were observed, slight differences in factors such as vaccination rates and age existed, potentially introducing bias. Not all carnival municipalities were included, and the distinction between carnival participants and non-participants was not possible, resulting in an ecological study. Classifying COVID-19 cases using data from test day and symptom onset introduces limitations to determining the exact peak on the number of infections. Lastly, there might have been bias due to a higher likelihood of individuals seeking testing post-carnival, potentially contributing to additional positive cases. Despite these limitations, we have no reason to assume they were of major impact on our conclusions.

In conclusion, this study underscores the influence of carnival on COVID-19 transmission, resulting in a notable increase in both cases and hospitalizations in the subsequent weeks. These findings underscore the importance of healthcare systems being prepared for potential rises in hospital admissions following similar large-scale events. In future pandemics, careful consideration should be given to additional measures, including event cancellations, to effectively mitigate transmission risks, particularly in the early stages of a pandemic.

During the preparation of this paper the author(s) used ChatGPT in order to streamline text and paragraphs and played a role in only the finalizing of the manuscript ( ± 10 % of the total time needed for this study). ChatGPT was not used in other parts of the research. After using this tool/service, the author reviewed and edited the content as needed and take(s) full responsibility for the content of the publication.

## Data availability statement

All data used in this paper is currently available from the RIVMdata website (https://data.rivm.nl/meta/srv/dut/catalog.search).

## Funding

There was no funding source for this study.

## Ethical statement

In the Netherlands, research is required to undergo review by an accredited Medical Research Ethics Committee if it is subject to the Dutch Medical Research Involving Human Subjects Act (WMO). All data presented in this paper is publicly available and anonymized. As such, our study does not fall under the scope of the WMO and therefore is exempt from medical ethical approval.

## Declaration of competing interest

None reported.
