# Supplementary material for: Impact of a large-scale event on SARS-CoV-2 cases and hospitalizations in the Netherlands, carnival seasons 2022 and 2023
Source: Public Health Pract (Oxf). 2024 Jun 27;8:100523. doi: 10.1016/j.puhip.2024.100523 (PMC11387216; doi:10.1016/j.puhip.2024.100523)
Supplement: Multimedia component 1 [file mmc1.pdf]

Supplementary material

Table S1 Aggregated weekly hospital admissions for both regions

Week number	Standardized hospitalizations carnival regions*	Standardized hospitalizations non-carnival regions*	Percentage increase in carnival regions
**8 (start of carnival)**	76.41	73.67	4%
**9 (end of carnival)**	86.03	72.02	19%
**10**	128.8	83.54	54%
**11**	145.39	98.68	47%
**12**	125.45	97.63	28%
**13**	97.11	78.65	23%
**14**	56.39	65.2	-14%
**15**	37.91	49.99	-24%
**16**	25.9	36.32	-29%
**17**	19.4	24.48	-21%
**Combined**	**798.79**	**680.18**	**17%**

*Per 1,000,000 inhabitants

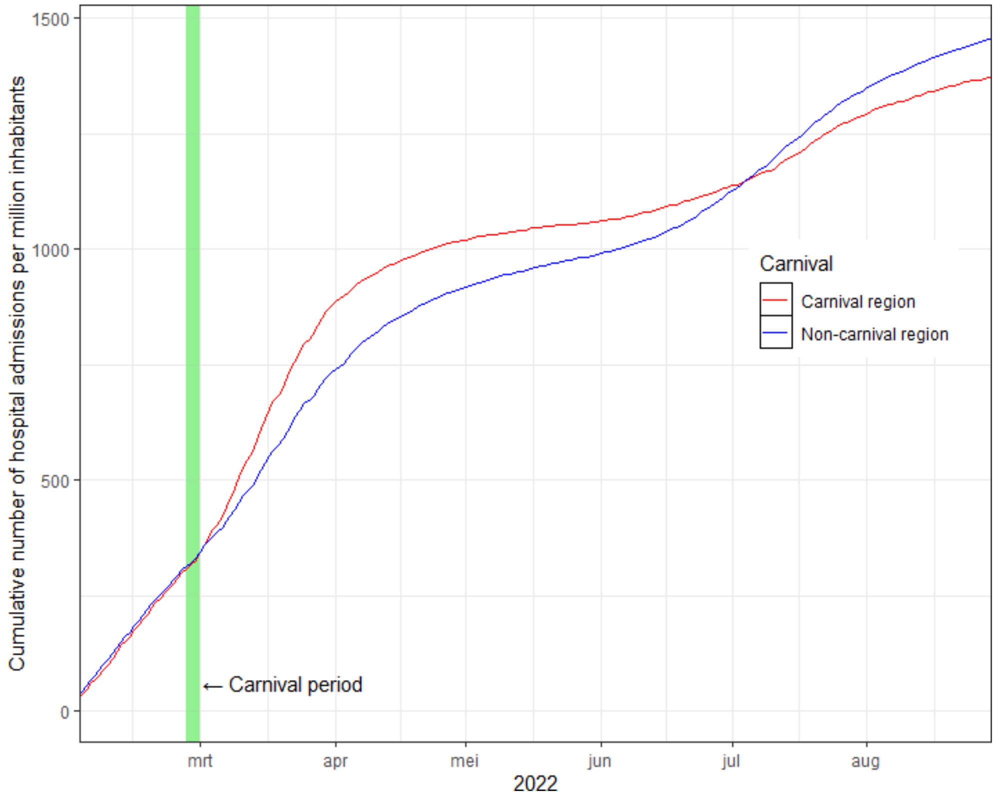

Figure S1 Cumulative hospital admissions 2022

Table S2 Aggregated weekly hospital admissions for both regions

Week number	Standardized hospitalizations carnival regions*	Standardized hospitalizations non-carnival regions*	Percentage increase in carnival regions
**7 (start of carnival)**	33.13	31.63	5%
**8 (end of carnival)**	62.51	36.6	71%
**9**	81.37	48.84	67%
**10**	78.41	51.44	52%
**11**	57.93	51.66	12%
**12**	37.45	45.6	-18%
**13**	30.98	35.09	-12%
**14**	18.86	27.3	-31%
**15**	14.82	21.4	-31%
**16**	9.7	20.89	-54%
**Combined**	425.16	370.45	15%

*Per 1,000,000 inhabitants

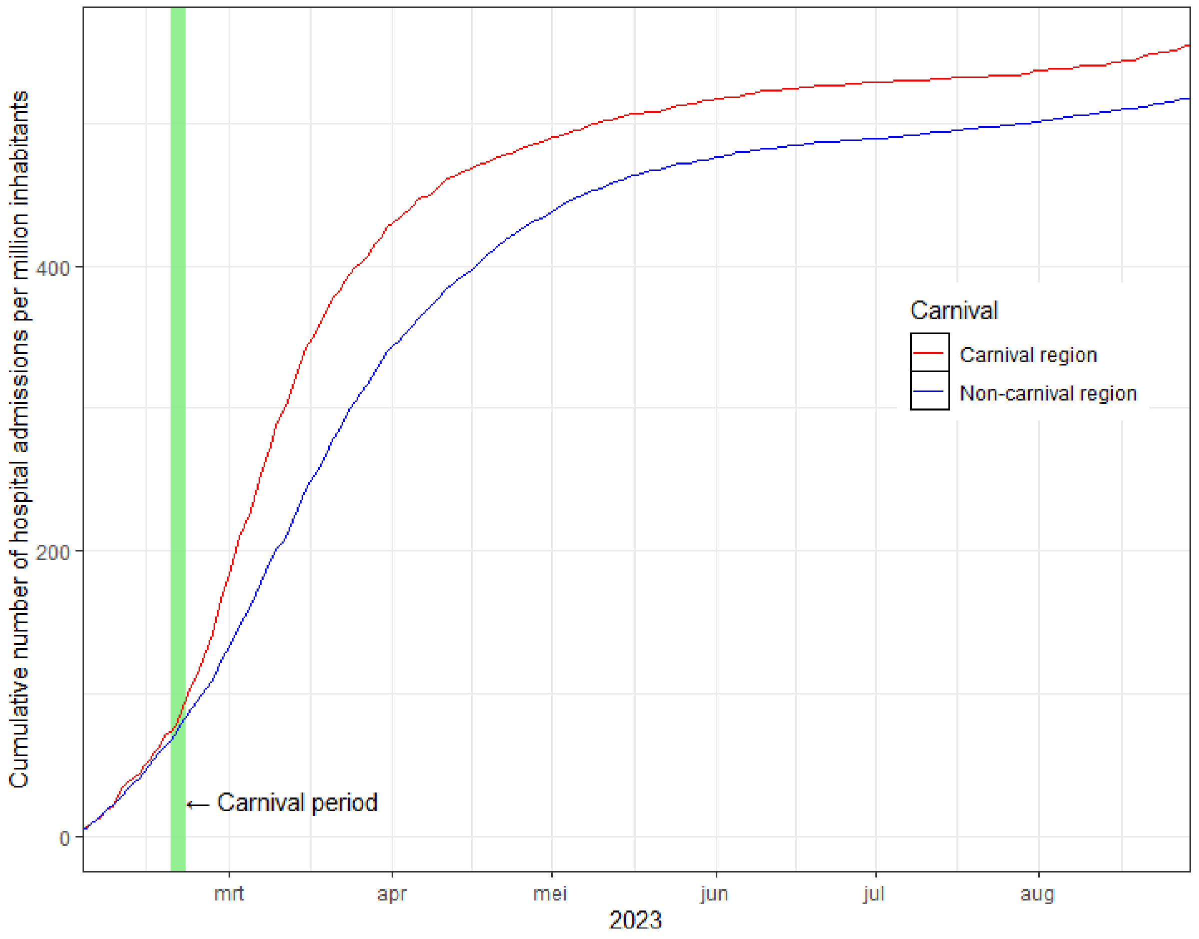

Figure S2 Cumulative hospital admissions 2023
